# The Thermodynamic and Kinetic Properties of the dA-rU DNA-RNA Hybrid Base Pair Investigated via Molecular Dynamics Simulations

**DOI:** 10.3390/molecules29204920

**Published:** 2024-10-17

**Authors:** Taigang Liu, Lei Bao, Yujie Wang

**Affiliations:** 1 School of Medical Engineering, Xinxiang Medical University, Xinxiang 453003, China; liuttgg@whu.edu.cn; 2 School of Public Health, Hubei University of Medicine, Shiyan 442000, China; bolly@whu.edu.cn; 3 Department of Physics and Telecommunication Engineering, Zhoukou Normal University, Zhoukou 466000, China

**Keywords:** DNA-RNA hybrid, base pair, thermodynamics, kinetics, molecular dynamics simulation

## Abstract

DNA-RNA hybrid duplexes play essential roles during the reverse transcription of RNA viruses and DNA replication. The opening and conformation changes of individual base pairs are critical to their biological functions. However, the microscopic mechanisms governing base pair closing and opening at the atomic level remain poorly understood. In this study, we investigated the thermodynamic and kinetic parameters of the dA-rU base pair in a DNA-RNA hybrid duplex using 4 μs all-atom molecular dynamics (MD) simulations at different temperatures. Our results showed that the thermodynamic parameters of the dA-rU base pair aligned with the predictions of the nearest-neighbor model and were close to those of the AU base pair in RNA. The temperature dependence of the average lifetimes of both the open and the closed states, as well as the transition path times, were obtained. The free-energy barrier for a single base pair opening and closing arises from an increase in enthalpy due to the disruption of the base-stacking interactions and hydrogen bonding, along with an entropy loss attributed to the accompanying restrictions, such as torsional angle constraints and solvent viscosity.

## 1. Introduction

DNA-RNA hybrids, consisting of a DNA strand and an RNA strand, are important intermediates in numerous biological processes such as Okazaki fragment formation during DNA replication and the reverse transcription process [1,2]. Around 59% of human genes contain sequences capable of forming such hybrids [3]. These hybrids play major roles in DNA replication, transcription, methylation, repair, telomerase replication, and reverse transcription [1,2,4,5,6,7,8,9,10], all of which rely on the formation and cleavage of these molecules. Recognized by RNase H, these hybrids can be specifically hydrolyzed on the RNA strand without affecting the complementary DNA strand [11,12]. Furthermore, several human diseases, including certain cancers and neurological disorders, are linked to the formation of DNA-RNA hybrids [13,14,15,16,17]. More recently, the application of gene editing techniques like CRISPR-Cas9 has sparked renewed interest in understanding DNA-RNA hybridization [18,19,20]. The biological functions of DNA-RNA hybrid duplexes are closely associated with their mechanical properties, particularly their kinetics and conformational changes. In the process of structural transitions, the formation and disruption of individual base pairs are key steps in many biological processes. Therefore, investigating the thermodynamic and kinetic properties of individual base pair opening and closing is crucial for a quantitative understanding of the biological functions of these hybrids.

The opening and flipping of the base pairs in RNA and DNA duplexes have been studied extensively using various experimental techniques, including NMR spectroscopy [21,22,23,24,25,26], fluorescence correlation spectroscopy (FCS) [27,28], and single-molecule forces [29]. However, limitations in these techniques leave some fundamental issues, such as the dynamics of single base pair opening and base flipping, unresolved. Therefore, all-atom molecular dynamics (MD) simulations have been widely employed to investigate the base pair opening in RNA and DNA duplexes [30,31,32,33,34,35,36,37,38,39,40,41,42,43,44]. For example, Pan et al. used MD simulations to study structural fluctuations in RNA and DNA duplexes, discovering that RNA base pairs opened into the major groove, whereas DNA showed no significant tendency for base pair opening [36]. Similarly, Zgarbova et al. explored base pair fraying in both DNA and RNA, observing that AT/AU terminal base pairs were significantly less stable than GC terminal pairs [43]. Quantitative studies have further explored the average lifetimes, barriers, and transition rates for forming and opening base pairs in both RNA and DNA duplexes [35,40]. In contrast, there has been less quantitative research, both experimentally and theoretically, on base pair opening in DNA-RNA hybrid duplexes. Experimental studies have demonstrated that the stability of hybrid base pairs differs from that of RNA and DNA base pairs. For instance, with the same base sequence in nucleic acid double helices, the stability of the rA-rU base pair is similar to that of the corresponding rA-dT base pair but differs from the stabilities of the dA-dT and dA-rU base pairs in the other two duplexes. Specifically, the dA-rU base pair is less stable than the rA-dT base pair, following the order: dA-rU < rA-rU < dA-dT [45,46,47]. The thermodynamic parameters of hybrid base pairs were first experimentally obtained by measuring the melting temperatures of hybrid duplexes [48], but these parameters were later revised due to their dependence on sequence and ion concentration [49]. Additionally, some fundamental problems, including the barrier of opening and forming the base pair and the quantitative results on the transition rates during base pair formation, remain unresolved.

In this study, using all-atom MD simulation, we quantitatively characterized the thermodynamic and kinetic parameters of the dA-rU base pair in DNA-RNA hybrid duplexes by directly simulating the opening–closing switch of the base pair. The computed results showed that the thermodynamic parameters of DNA-RNA hybrid duplex base pairs were consistent with those of the nearest-neighbor model [49] and close to the thermodynamic parameters of the AU base pair in RNA [40]. Additionally, the temperature dependence of the transition rates for the base pair’s opening and closing processes was obtained.

## 2. Results and Discussion

### 2.1. Definition of Closed State (cs), Open State (os), and Transition State (ts)

**Figure 1:**
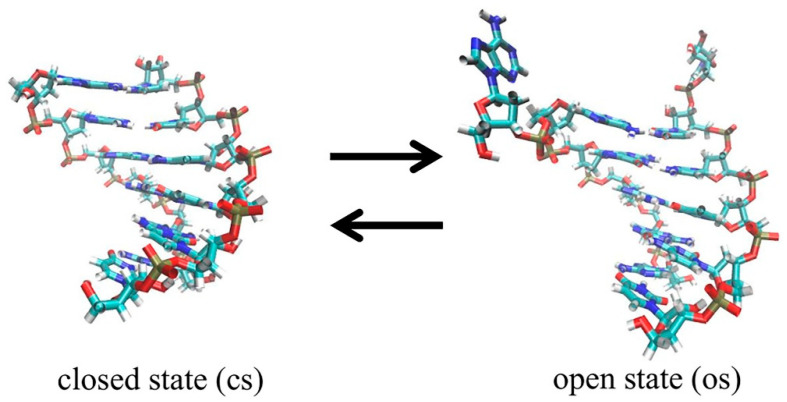
The two typical structures of a DNA-RNA hybrid with closed and open terminal base pairs: (**left**), closed state; (**right**), open state.

**Figure 2:**
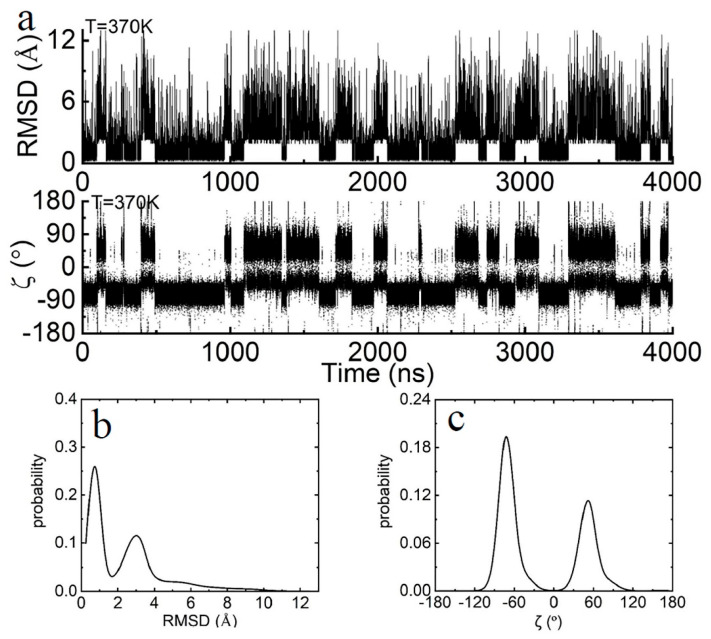
(**a**) The RMSD values and torsional angles ζ for the dA-rU base pair over the entire simulation period at 370 K. (**b**) The distribution of the RMSD of the dA-rU base pair over the entire simulation period at 370 K. (**c**) The distribution of the torsional angles ζ of the dA-rU base pair over the entire simulation period at 370 K.

As in our previous analyses [35,40,41,42], the terminal base pair dA-rU would undergo the closing–opening transition process (see Figure 1) through an intermediate transition state. The corresponding conformations can be categorized into three states: open, closed, and transition. These states are identified based on the time-dependent root mean square deviation (RMSD) for the terminal bases dA and rU, relative to their initial structure, as well as the backbone torsion angle ζ. This torsion angle refers to the dihedral angle formed by the four atoms C3′(i)-O3′(i)-P(i+1)-O5′(i+1), where i represents the ith nucleotide in a polynucleotide chain, as shown in Figure 2.

**Figure 3:**
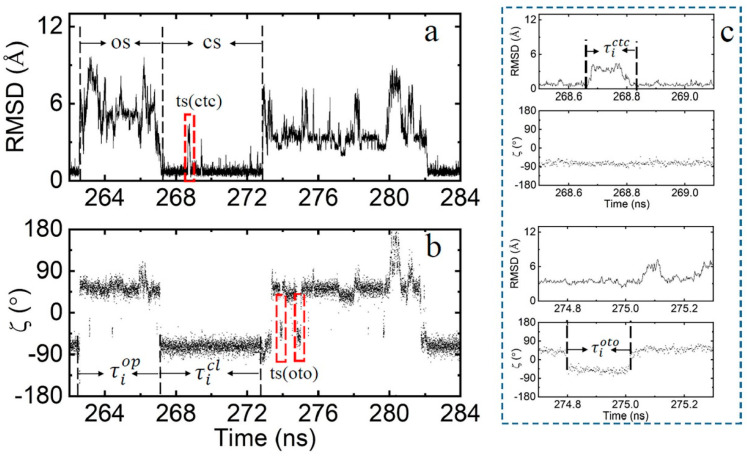
(**a**) The RMSD values and (**b**) torsional angles ζ for the simulation period from 262 to 284 ns at 370 K. (**c**) The RMSD values and torsional angles ζ near the transition states at 370 K.

For the closed state, the RMSD value is centered around 0.7 Å and the torsion angle ζ is approximately −75° (−50° to −100°), as shown in Figure 2. In this conformation, the two terminal bases, dA and rU, exhibit slight vibrations around their initial positions, with the base pairing and the base-stacking interactions with neighboring nucleotides remaining largely intact. In the open state, the RMSD value varies from 2 to 13.5 Å, and the torsion angle ζ centers around 50°, as shown in Figure 2. In this conformation, the two terminal bases, dA and rU, shift away from their initial positions and flip into the solvent, leading to the disruption of base-pairing and base-stacking interactions. For the transition state, the RMSD value exceeds 2 Å and has a very short residence time, although the torsion angle ζ remains within the closed state region. Simultaneously, the terminal bases flip outward into the solvent. Depending on the transition pathway, the transition state can be further classified into two types, “ctc” (transitioning from the closed state and returning to the closed state) and “oto” (transitioning from the open state and returning to the open state), as shown in Figure 3.

### 2.2. Thermodynamic Properties of the Terminal dA-rU Base Pair

**Figure 4:**
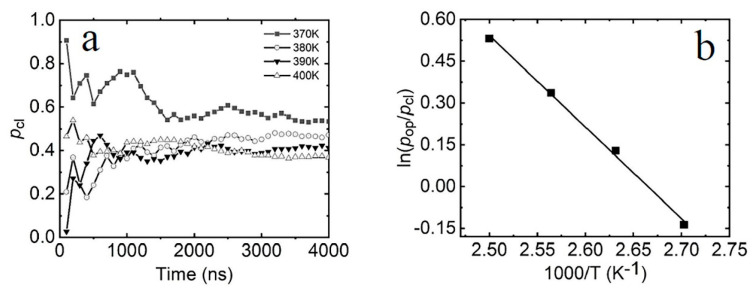
(**a**) The probability in the closed state over the simulation time for each temperature. (**b**) The temperature dependence of ln(*p*_op_/*p*_cl_) at 370 K, 380 K, 390 K, and 400 K. Line: line fit; square: simulation results.

At each simulation temperature, the population distributions of the closed, open, and transition states were calculated using the Formula (1)

(1) $$p_{cl}=\tau_{cl}/\tau =\sum_{i=1}^{N_{cl}}\tau_{i}^{cl}/\tau ,\;\;{\;p}_{op}=\tau_{op}/\tau =\sum_{i=1}^{N_{op}}\tau_{i}^{op}/\tau ,\;\;\;\;p_{ts}=\tau_{ts}/\tau =\sum_{i=1}^{N_{ts}}\tau_{i}^{ts}/\tau$$

where τ is the total simulation time and $\tau_{cl}$, $\tau_{op},$ and $\tau_{ts}$ represent the total time spent in the closed, open, and transition states, respectively. $N_{cl}$, $N_{op}$, and $N_{ts}$ denote the total number of snapshots in which the corresponding conformations reside in the closed, open, and transition states. $\tau_{i}^{cl}$, $\tau_{i}^{op}$, and $\tau_{i}^{ts}$ refer to the *i*th duration the conformations remain in the respective state. The probability of occupying the closed state at different temperatures during the simulation is shown in Figure 4a. Each point represents the probability calculated over time intervals from the beginning up to the respective simulation time. When the simulation time exceeds 3500 ns, the occupied probability of the closed state has a stable value. To ensure that the system reaches equilibration between the open and closed states, the simulation time is set to 4000 ns for each temperature. The probabilities of the closed, open, and transition states at the end of simulations are provided in Table 1.

Since the probabilities of the closed and open states are much higher than those of the transition states, the system was initially modeled using a simple opening–closing two-state model. According to the equilibrium probability distribution, the probability of each conformation among all base pairs in the hybrid can be calculated using the equation $p_{i}=\exp(-G_{i}/k_{B}T)/\sum_{j}\exp(-G_{j}/k_{B}T)$, where $G_{i}$ is the free energy of the *i*th conformation when the system reaches equilibrium, $k_{B}$ is the Boltzmann constant, *T* is the absolute temperature, and $\sum_{j}\exp(-G_{j}/k_{B}T)$ is the partition function. The free-energy differences (ΔG) between the two states can then be determined as (2)

(2) $$\Delta G=-k_{B}Tln(\frac{p_{op}}{p_{cl}})$$

where *p*_op_ and *p*_cl_ represent the probabilities of the open and closed states, respectively; $k_{B}$ is the Boltzmann constant, and *T* is the absolute temperature. As shown in Figure 4b, the free-energy differences (ΔG) exhibit a linear relationship with the reciprocal of temperature (1/T). Using the Gibbs free-energy equation ΔG=ΔH−TΔS, where ΔH and ΔS are the enthalpy and entropy changes during the transition between the two states, the thermodynamic parameters of the terminal dA-rU base pair in hybrid duplexes were determined: ΔH=−6.83 kcal/mol and ∆S=−18.2 eu. These values are consistent with those predicted by the nearest-neighbor model [49] and are close to the thermodynamic parameters (ΔH=−7.3 kcal/mol, ∆S=−18.5 eu) [40] for the AU base pair in RNA. These findings also align well with experimental data on thermal stability [45,46].

### 2.3. The Kinetic Mechanism of the Terminal dA-rU Base Pair

**Figure 5:**
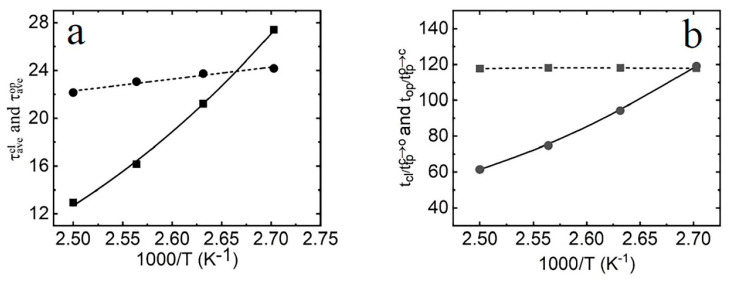
(**a**) Temperature dependence of the average lifetime of the closed state (square) and the open state (circle). Lines: fitted with Equation (3); symbols: MD simulations. (**b**) Temperature dependence of the ratios of $t_{cl}/t_{tp}^{c\to o}$(circle) and $t_{op}/t_{tp}^{o\to c}$ (square). Lines: fitted with Equations (3) and (4); symbols: MD simulations.

The average lifetimes of the closed, open, and transition states can be calculated through $\tau_{ave}=\sum_{i=1}^{N}\tau_{i}/N$, where $\tau_{ave}$ is the average lifetime, $\tau_{i}$ is the *i*th lifetime of the conformation in its *i*th occurrence, and *N* is the total number of occurrences of the base pair in each state. The average lifetimes of the three states are listed in Table 1. It is clear that the average lifetime of the transition state is significantly shorter than that of the closed and open states. In the context of the opening–closing two-state model, the opening rate $k_{-}$ (from the closed state to the open state) and the closing rate $k_{+}$ (from the open state to the closed state) can be obtained using the formulas $k_{-}=1/\tau_{ave}^{cl}$, $k_{+}=1/\tau_{ave}^{op}$, where $\tau_{ave}^{cl}$ is the average lifetime of the closed state and $\tau_{ave}^{op}$ is the average lifetime of the open state. Figure 5a illustrates the temperature dependence of the average lifetime of the closed and open states. It can be observed that the average lifetime of the closed state exhibits a strong dependence on temperature, while that of the open state shows only a weak temperature sensitivity. This finding is consistent with both theoretical results [35,40,41,42] and the experimental findings [50] that the folding and unfolding rates have different sensitivities to temperature.

Under the three-state model (closed, open, and transition states), the transition rate from the transition state to the open state, and $k_{t\to o}$, is equal to the reciprocal of the average lifetime of the transition state oto: $k_{t\to o}=1/\tau_{ave}^{oto}$. Similarly, the transition rate from the transition state to the closed state, $k_{t\to c}$, is given by $k_{t\to c}=1/\tau_{ave}^{ctc}$. The transition path time from the open state to the closed state, $t_{tp}^{o\to c}$, and from the closed state to the open state, $t_{tp}^{c\to o}$, can be expressed as $t_{tp}^{o\to c}=\tau_{ave}^{ctc}$ and $t_{tp}^{c\to o}=\tau_{ave}^{oto}$. During the transition between the closed and open states of the base pair, the transition path times are much shorter than the lifetimes of the closed and open states and show only weak temperature-dependent behavior (see Table 1). Based on the transition-state theory [51,52,53,54,55,56], the average residence time in the open (*t*_op_) (3) state, closed (*t*_cl_) (4) state, and the transition path time $t_{tp}^{o\to c}$ (5) and $t_{tp}^{c\to o}\;(6)$, can be calculated as follows:

(3) $$t_{op}={\frac{1}{k}}_{+}=\frac{2\pi }{\beta D^{*}\omega^{*}\omega_{o}}\exp\left(\beta \Delta G_{o}\right)\;$$

(4) $$t_{cl}={\frac{1}{k}}_{-}=\frac{2\pi }{\beta D^{*}\omega^{*}\omega_{c}}\exp\left(\beta \Delta G_{c}\right)\;$$

(5) $$\;t_{tp}^{o\to c}=\frac{1}{\beta D^{*}(\omega^{*}{)}^{2}}\ln\left(2e^{\gamma }\beta ∆G_{o}\right)\;$$

(6) $$t_{tp}^{c\to o}=\frac{1}{\beta D^{*}(\omega^{*}{)}^{2}}\ln\left(2e^{\gamma }\beta ∆G_{c}\right)$$

where $\beta =1/k_{B}T$, with $k_{B}$ being the Boltzmann constant and *T* the absolute temperature. $D^{*}$ represents the diffusion coefficient at the top of the free-energy barrier; ${\left(\omega_{o}\right)}^{2}$, ${\left(\omega_{c}\right)}^{2}$, and ${\left(\omega^{*}\right)}^{2}$ refer to the curvatures of the free-energy surface in the open state, the closed state, and at the barrier, respectively. γ is Euler’s constant, $∆G_{o}$ represents the free-energy barrier height from the open state to the closed state, and $∆G_{c}$ represents the free-energy barrier height from the closed state to the open state. Equations (3) and (4) are attributed to Kramers [53], while Equations (5) and (6) are derived from Szabo [54,55]. Szabo’s equation makes the same assumptions and approximations as Kramers’ with regard to the underlying physics. The major distinction between Kramers’ theory and the transition state theory lies in the fact that the pre-exponential factor of the latter does not contain a diffusion coefficient and is simply 2π/ω, where ω can represent either $\omega_{o}$ or $\omega_{c}$.

According to Equations (3), (4), (5) and (6), the ratios of $t_{cl}/t_{tp}^{c\to o}$ and $t_{op}/t_{tp}^{o\to c}$ are dependent only on $\omega^{*}/\omega_{c}$, $\omega^{*}/\omega_{o}$ and the height of the free-energy barrier, but are independent of the diffusion coefficient $D^{*}$. As shown in Figure 5b, $t_{op}/t_{tp}^{o\to c}$ is nearly temperature-independent. Given that $\omega^{*}/\omega_{o}$ is constant, similar to protein folding [52], the free-energy barrier for the transition from the open state to the closed state should be temperature-dependent, implying that $∆G_{o}\propto T$. By fitting the two curves, the free-energy barrier of the base pair $∆G_{c}$ from the closed state to the open state is calculated to be 6.83 kcal/mol, which aligns with the magnitude of enthalpy change ∆H from the open state to the closed state. Based on the free-energy change between the open and the closed states, $∆G=∆G_{c}-∆G_{o}=∆H-T∆S$, the free-energy barrier for opening the base pair from the closed state is $∆G_{o}=T∆S$, where ∆S is the entropy change between the closed and open states. This indicates that breaking a single base pair is primarily driven by an increase in enthalpy, Δ*H*, due to the disruption of base-stacking interactions and hydrogen bonding. Conversely, forming a single base pair mainly involves the entropy loss, Δ*S*, arising from factors such as the conformation restriction required for pairing with each other, solvent viscosity, and other influences.

## 3. Materials and Methods

The initial structure for the 6 bp helix with the sequence $\left(\begin{array}{c} 5^{\prime }-dAAGAGA-3^{\prime }\\ 3^{\prime }-rUUCUCU-5^{\prime } \end{array}\right)$ was obtained from its crystal structure (PDB ID: 1DRR). The structure was immersed in a TIP3P [57,58] water box with a water shell of 15 Å in a triclinic box measuring 5.5 × 5.3 × 5.3 nm. The system was subsequently neutralized by the addition of 12 Na^+^ counterions, and additional Na^+^ and Cl^−^ ions were randomly placed to maintain 0.5 M NaCl concentration. The final system included approximately 15,326 atoms. The solvent and counterions were relaxed by energy minimization to equilibrium for 60 ns at 298 K. One of the equilibrium structures was then selected as the starting structure for further simulations at high temperatures.

All simulations were performed using the GROMACS 4.6.7 simulation package [59]. The amber force fields for DNA and RNA were Amber bsc1 and χOL3 [60,61], respectively. Periodic boundary conditions were employed. Temperature coupling was handled by velocity rescaling [62], and pressure coupling used the Parrinello–Rahman barostat algorithm [63]. Electrostatic interactions were evaluated via the Particle-Mesh Ewald (PME) method [64] with a 10 Å direct space cutoff, while Lennard-Jones interactions [65] were truncated at 10 Å. Bond lengths involving hydrogen atoms were constrained using the LINCS algorithm [66], and water molecules were kept rigid using the SETTLE algorithm [67]. The neighboring grid search method was applied and updated every 10 steps. The equations of motion were integrated using the Verlet algorithm with a 2 fs time step, and the coordinates were saved every 2 ps. To specifically investigate the thermodynamic and kinetic properties of the opening and closing of the terminal dA-rU base pair with the nearest neighbors and next-nearest neighbors $\left(\begin{array}{c} 5^{\prime }-dAAG\cdots \\ 3^{\prime }-rUUC\cdots \end{array}\right)$ in the DNA-RNA hybrid, all atoms of DNA-RNA hybrid duplexes except the two terminal nucleotides 5′-dA and 3′-rU were fixed using a harmonic potential with a force constant of 2000 kJ/mol·nm^−2^. To accelerate equilibration, the simulation temperatures were set at 370 K, 380 K, 390 K, and 400 K, where the population of the open and closed states each occupied approximately 50%.

## 4. Conclusions

In summary, through microsecond (4 μs) all-atom molecular dynamics (MD) simulations conducted at various temperatures, the thermodynamic and kinetic parameters of the dA-rU base pair in the DNA-RNA hybrid duplex were derived. Our calculations showed that the entropy change ∆*S* and the enthalpy change ∆*H* for opening/closing the dA-rU base pair were consistent with the nearest-neighbor model. These results were close to the thermodynamic parameters of the AU base pair in RNA, aligning well with the experimentally measured single base pair stabilities in RNA and DNA-RNA hybrid duplexes. The average lifetimes of the open state and the closed state showed different dependence on temperature, while the transition path times exhibited only weak temperature dependence. The breaking of a single base pair results from the enthalpy increase ∆*H* caused by the disruption of the base-stacking interactions and hydrogen bonds. Conversely, the formation of a single base pair is driven primarily by the unfavorable entropy loss ∆*S* due to accompanying restrictions, such as the torsional angles and solvent viscosity. In conclusion, our simulations provide an atomic-level insight into the opening–closing transition of the dA-rU base pair within a DNA-RNA hybrid duplex and reveal the microscopic mechanism governing the behavior of individual dA-rU base pairs.

## Figures and Tables

**Table 1:** The average lifetime *τ*_ave_ (ns) and the total number N of occurrences of conformations at closed, open, and transition states at different temperatures (K) during the 4000 ns simulation.

Temperature	Closed State (cs)			Open State (os)			Transition State (ctc)			Transition State (oto)		
*T*(K)	$\tau_{ave}^{cl}$(ns)	*N*_cl_	*p*_cl_	$\tau_{ave}^{op}$(ns)	*N*_op_	*P*_op_	$\tau_{ave}^{ctc}$(ns)	*N*_ctc_	*P*_ctc_	$\tau_{ave}^{oto}$(ns)	*N*_oto_	*P*_oto_
370	27.41	78	0.53	24.18	77	0.47	0.23	435	0.025	0.205	663	0.034
380	21.21	89	0.47	23.74	89	0.53	0.225	441	0.025	0.201	736	0.037
390	16.16	102	0.41	23.06	102	0.59	0.216	447	0.024	0.195	762	0.037
400	12.95	114	0.37	22.14	114	0.63	0.211	462	0.023	0.188	821	0.039

## Data Availability

Data are contained within the article.
